# Record of occurrence and histopathological changes induced by nematodes of the family Capillariidae in the tongue of fish, reptiles, birds, and mammals

**DOI:** 10.29374/2527-2179.bjvm003826

**Published:** 2026-05-14

**Authors:** Luís Eduardo Seabra de Freitas, Lucas Araújo Ferreira, Elaine Lopes de Carvalho, Elane Guerreiro Giese

**Affiliations:** 1 Laboratório de Histologia e Embriologia Animal. Instituto da Saúde e Produção Animal. Universidade Federal Rural da Amazônia (LHEA/ISPA/UFRA). Belém, PA, Brazil.

**Keywords:** parasites, helminths, *Capillaria* sp., *Eucoleus* sp., histopathology, parasitos, helmintos, *Capillaria* sp., *Eucoleus* sp., histopatologia

## Abstract

The family Capillaridae comprises of nematodes within the order Trichinellida that parasitize a wide range of vertebrate hosts, targeting the gastrointestinal, respiratory, and urinary tracts. While these helminths are frequently recorded in scientific literature, few studies report their presence in other anatomical sites such as the tongue. With that in mind, this study provides a systematic review of articles from 1975 to January 2026 focusing on the description of the presence and pathological alterations caused by helminths from the family Capillariidae whose habitat is the tongue of animals A comprehensive search was conducted across multiple databases, including SciELO, PubMed, BVS, Periódico Capes, and ACS publications platforms, using the descriptors “Tongue AND Capillariidae;” “Tongue AND *Eucoleus*,” “Tongue AND *Capillaria*,” and “Histopathology AND Capillariidae”. Of the 83 studies initially identified, 18 met the inclusion and exclusion criteria for analysis, and were used in the present study. The review identified 2 genera *Eucoleus* and *Capillaria*, parasitizing the tongue. These parasites were found embedded in the stratified squamous keratinized epithelium of the tongue, with adult helminths most frequently localized in the caudal third of the tongue, with eggs being present. In most cases, no significant morphological abnormalities; however, in the case of *E. garfiai*, was described that showed the presence of adjacent lymphocytic and eosinophilic inflammatory infiltrate, and hyperkeratosis caused by the helminth. Given the limited number of detailed reports, further research is needed to better characterize tissue inflammatory reactions and elucidate the potential clinical and pathological implications of lingual capillariasis.

## Resumo

A família Capillariidae compreende nematoides da ordem Trichinellida que parasitam uma ampla gama de hospedeiros vertebrados, afetando os tratos gastrointestinal, respiratório e urinário. Embora esses helmintos sejam frequentemente registrados na literatura científica, poucos estudos relatam sua presença em outros sítios anatômicos, como a língua. Com isso em mente, este estudo apresenta uma revisão sistemática de artigos publicados entre 1975 e janeiro de 2026, com foco na descrição da presença e das alterações patológicas causadas por helmintos da família Capillariidae, cujo habitat é a língua de animais. Os descritores utilizados foram: “Língua e *Eucoleus*”, “Língua e *Capillaria*” e “Histopatologia e Capillariidae”. Dos 83 estudos inicialmente identificados, 18 atenderam aos critérios de inclusão e exclusão para análise e foram utilizados no presente estudo. A revisão identificou dois gêneros, *Eucoleus* e *Capillaria*, que parasitam a língua. Esses parasitos foram encontrados incrustados no epitélio escamoso estratificado queratinizado da língua, com os helmintos adultos localizados mais frequentemente no terço caudal da língua, onde também estavam presentes os ovos. Na maioria dos casos, não foram observadas anormalidades morfológicas significativas, entretanto, no caso de *E. garfiai*, foi descrita a presença de infiltrado inflamatório linfocítico e eosinofílico adjacente, além de hiperceratose causada pelo helminto. Devido ao número limitado de relatos detalhados, são necessárias mais pesquisas para melhor caracterizar as reações inflamatórias teciduais e elucidar as potenciais implicações clínicas e patológicas da capilariose lingual.

## Introduction

Capillariidae (Railliet, 1915) is a family of helminths in the phylum Nematoda, order Trichinellida, which has more than 20 genera and 400 species with a worldwide distribution. This family is characterized by filiform, delicate, and elongated nematodes that can parasitize various host systems, including the respiratory, gastrointestinal, and urinary tracts. Parasitism by the genera *Capillaria* and *Eucoleus* is frequent in the gastrointestinal system, especially in the esophagus, where the largest number of these helminths occur (Calvani et al., 2021; Tamaru et al., 2025).

These genera of helminths are found across a wide range of vertebrate hosts, including fish, reptiles, amphibians, birds, and mammals. Because their life cycles are generally monoxenic, infection typically begins when a host ingests capillariid eggs, either through predation or by scavenging the remains of another animal. Throughout the parasite’s life cycle, the tongue can be a site of infection; the nematode may be acquired through the act of feeding or via social behaviors such licking the genitals or anus of other animals (Nunes, 2019; Pinheiro et al., 2018).

Despite the diversity of capillariids that parasitize various hosts, there is a limited number of studies that address the presence of this family on animal tongues. With a that in mind, the present work aimed to perform out a systematic literature review on articles focusing describing the presence and morphological traces of the parasite-host relationship of helminths of the Capillariidae family on the tongue of animals.

## Materials and methods

### Type of study

This is a qualitative and quantitative systematic review that focuses on the description of helminths of the family Capillariidae found in the component tissues of animal tongues from 1980 to July 2025. For the study design, the recommendations of the PRISMA 2020 guidelines were adopted and adapted, as described by Page et al. (2021) and Ferreira et al. (2025), to ensure greater transparency in the methodology employed and the results obtained.

### Descriptors and databases

The active search was conducted using articles in all languages on the Scientific Electronic Library Online (SciELO), National Library of Medicine (PubMed), and Virtual Health Library (BVS) platforms, CAPES Journals, and American Chemical Society (ACS publications), adopting the combination of English descriptors registered in BVS: Tongue, Capillariidae, *Eucoleus*, *Capillaria*, and Histopathology. The English terms were combined using the "AND" operator, which limits the search results to documents containing all the specified descriptors, thus optimizing the search.

### Inclusion and exclusion criteria

Studies published between 1975 and January 2026 were included that reported C*apillaria* identified from eggs in collected feces or adult nematodes recovered from necropsies of animals submitted to laboratory examinations. Articles that fell outside the study theme, duplicates, and studies that failed to identify the nematode family or group were excluded, along with review articles, notes that did not include the scope of the research, comments, or incomplete results.

### Search strategy

Data were collected between February and March 2026. The initial evaluation of the bibliographic material was conducted by reviewing titles and abstracts and selecting those aligned with study objectives. Subsequently, a complete reading and classification of the selected articles was performed based on the inclusion and exclusion criteria mentioned above. The data were recorded in Microsoft Excel 16 spreadsheets and used to construct figures, graphs, and tables of interest. Additionally, two external reviewers reanalyzed all the data.

### Summary of results

**Figure 1:**
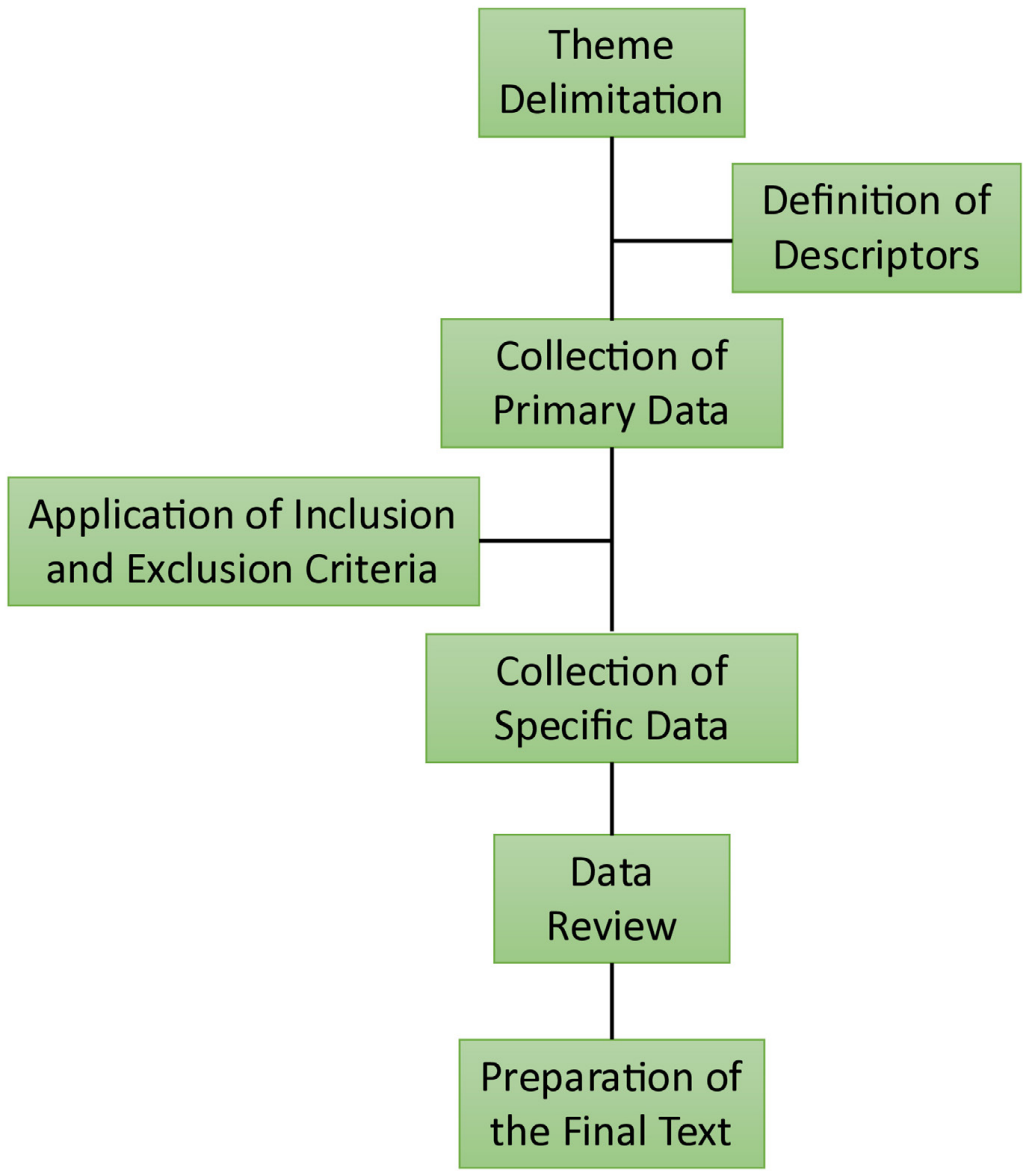
Flowchart of the main stages and structuring and analysis of the research.

The results of the database search were organized into a flowchart. After review by independent researchers, the articles considered suitable for inclusion in the research were categorized by the diagnostic methods used to detect infection in hosts, including eggs and adult worms. The following details were documented: year of publication, authors, host country, host, and reported parasites. In addition, figures were created to illustrate the distribution of recorded cases at the global and national levels, offering a better understanding of the interest in the topic. The final text was compiled by critically comparing the main results of each study and incorporating all relevant articles for discussion and analysis. The information was organized according to the helminth genus, host, and country of origin. The research steps and workflow were organized in a way that allows for an understanding of how the research was conducted, as shown in Figure 1.

## Results

**Figure 2:**
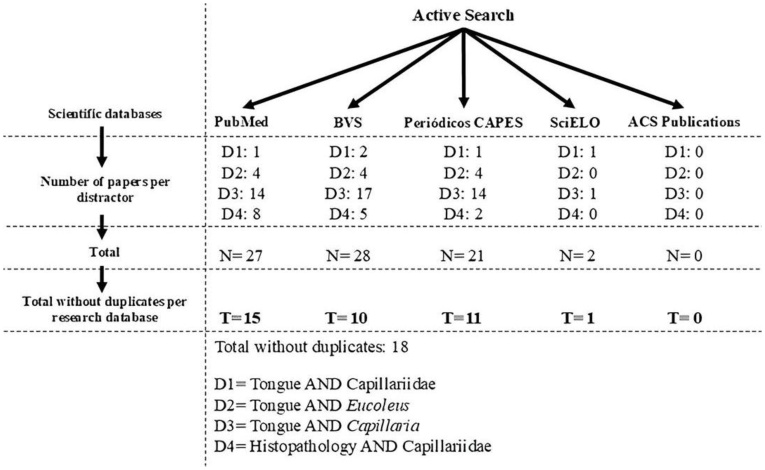
Flowchart for obtaining articles from the databases PubMed, BVS, ACS publication, CAPES Periódicos and SciELO.

The active search, conducted over almost 50 years, resulted in 83 articles in the five databases within the stipulated period. Of these, 32 were found in PubMed, 30 in BVS, 0 in ACS publications, 22 in CAPES Journals, and 1 in SciELO. After applying the inclusion and exclusion criteria, 45 studies were approved for the study. Excluding duplicates, 18 studies comprised the present study, as shown in Figure 2.

**Figure 3:**
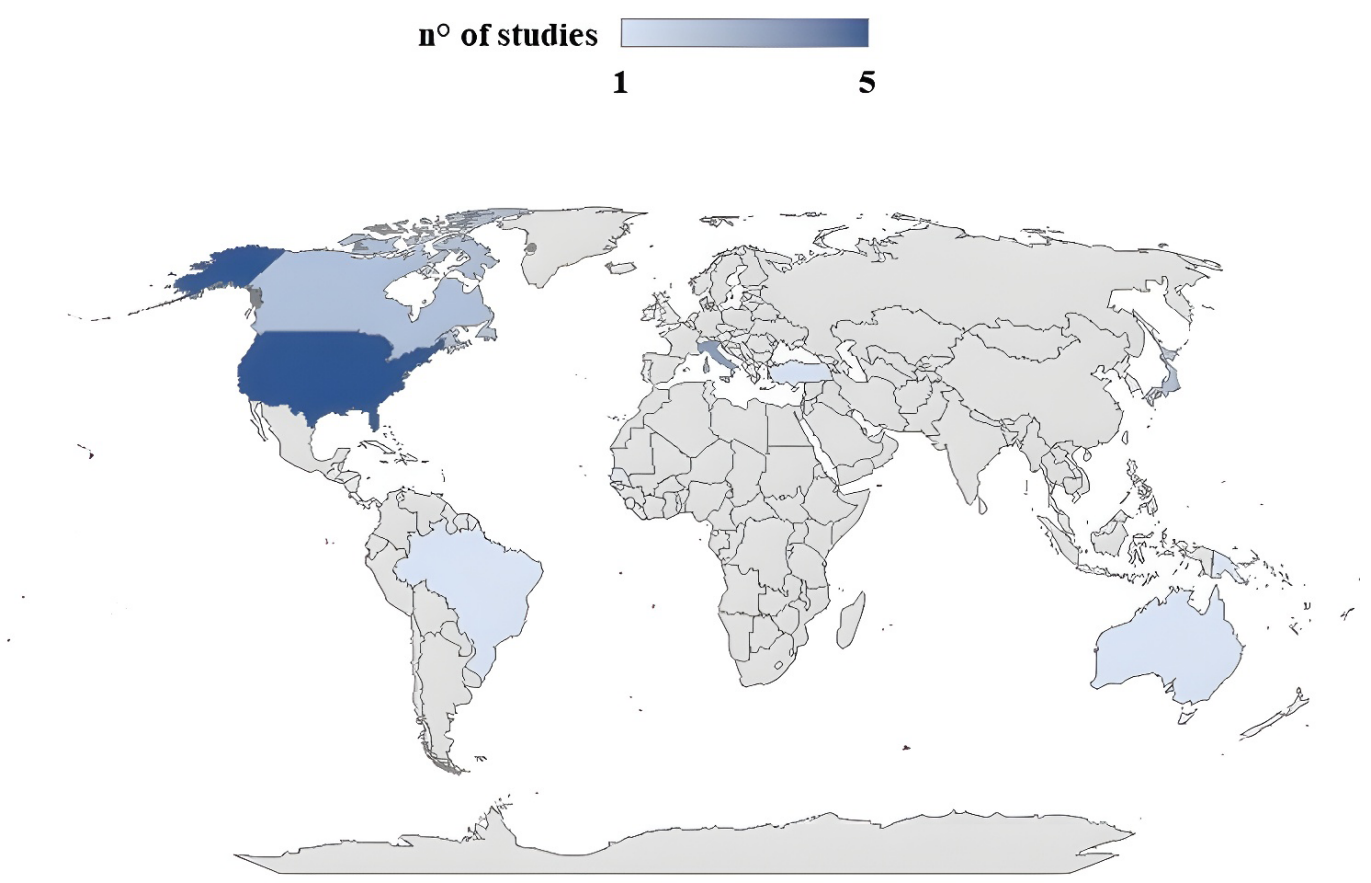
Map showing the number of studies on the topic worldwide, with the main records concentrated in North America between the years 1975 and January 2026.

The 18 studies analyzed revealed cases distributed worldwide in countries such as Papua New Guinea, Senegal, Türkiye, United States of America (USA), Brazil, Canada, Italy, Japan, and Australia, as shown in Figure 3.

**Figure 4:**
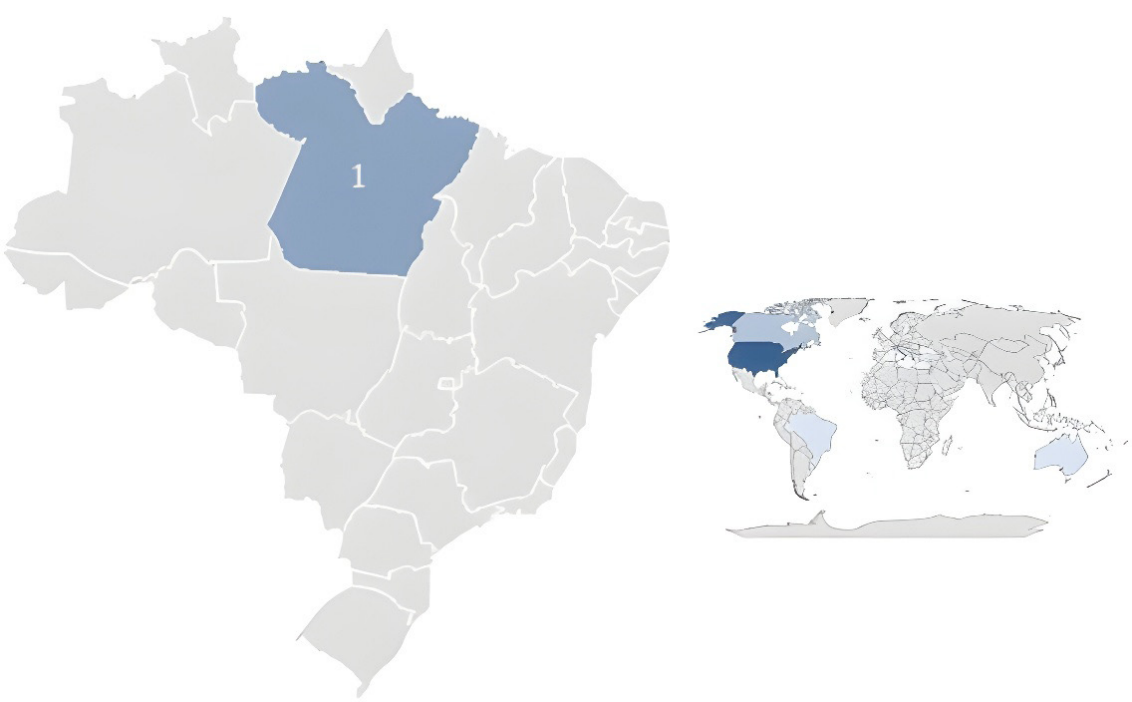
Map showing the number of studies on the topic in Brazil between the years 1975 and January 2026.

Of all the studies analyzed, only 1 case was reported in Brazil, specifically in the city of Paragominas, in the state of Pará, as shown in Figure 4.

**Figure 5:**
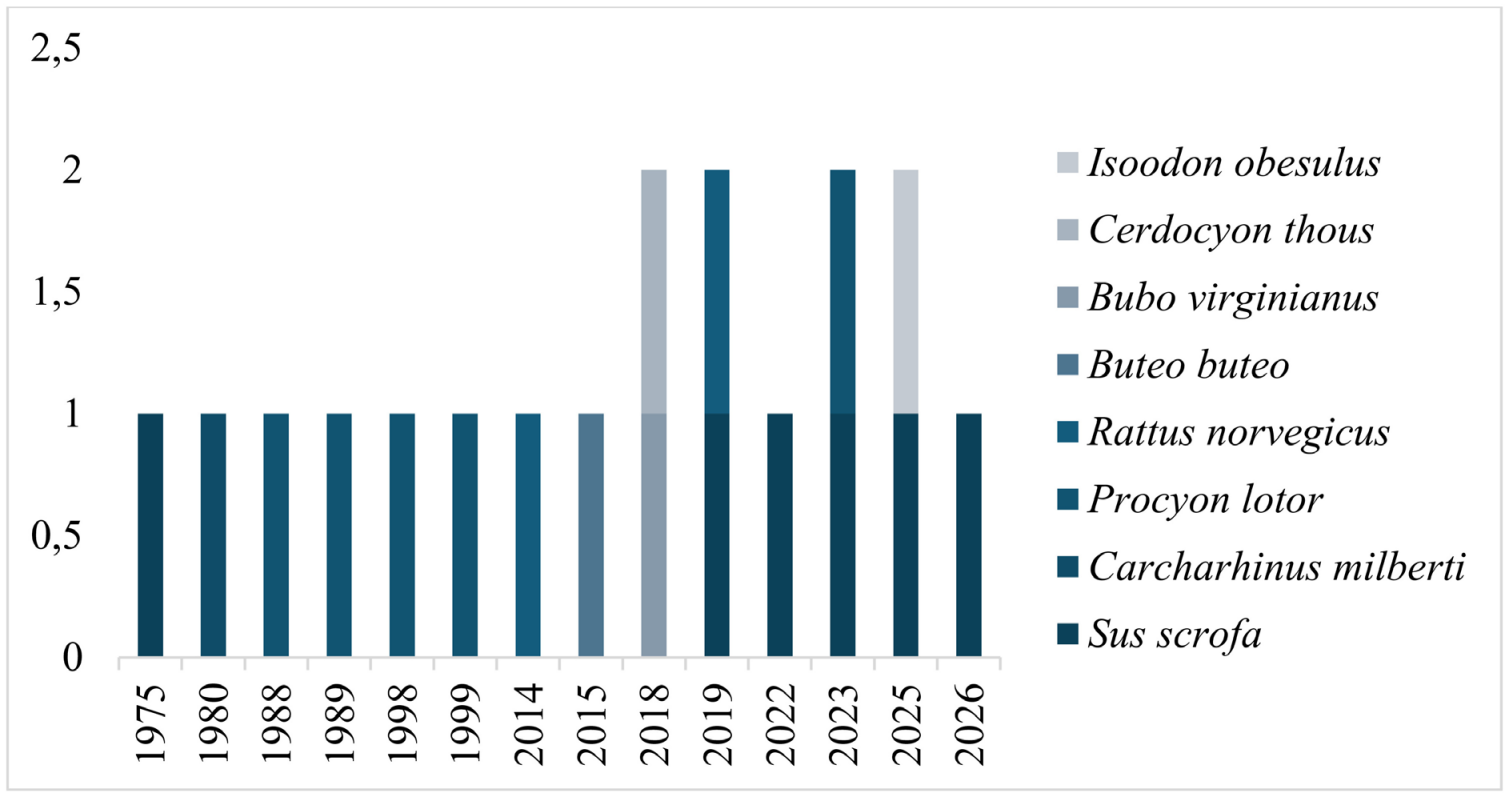
Graph showing the number of studies for each host of Capillariidae found on the tongue.

After screening, the studies were organized according to the year of publication and the hosts parasitized by the Capillaridae family, as shown in Figure 5.

The articles were identified by various diagnostic techniques, such as necropsy, conventional microscopy, scanning electron microscopy (SEM), histology, or molecular techniques, as shown in Table 1, which presents the following information: year of publication (ascending order), authors, hosts parasitized, country of origin, parasites, technique, and parasitic form found.

**Table 1:** Number of published works on the presence of Capillaridae in the tongue of animals between 1975 and July 2025 found in the PubMed, BVS, ACS publications, CAPES Periódicos and SciELO databases.

**Nº**	**Authors**	**Host**	**Country**	**Parasite**	**Technique**	**Form found**
1	Copland (1975)	*Sus scrofa*	Papua New Guinea	*Capillaria papuensis*	Histology	Adults and Eggs
2	Van Banning (1980)	*Cynoglossus browni*	Senegal	*Capillaria spinosa*	Necropsy	Eggs and Larva
3	Snyder (1988)	*Procyon lotor*	USA	*Capillaria procyonis*	Necropsy	Eggs
4	Snyder (1989)	*Procyon lotor*	USA	*Capillaria procyonis*	Histology	Adults and Eggs
5	Hamir & Rupprechtt, (1998)	*Procyon lotor*	USA	*Capillaria* sp.	Histology	Adults
6	Hamir & Snyder (1999)	*Procyon lotor*	USA	*Capillaria* sp.	Histology	Adults and Eggs
7	Rothenburguer et al. (2014)	*Rattus norvegicus*	Canada	*Eucoleus* sp*.*	Histology	Eggs
8	Yildiz et al. (2015)	*Buteo buteo*	Türkiye	*Eucoleus dispar*	Histology	Adults and Eggs
9	Royal & Jochems (2018)	*Bubo virginianus*	USA	Capillariidae	Swab impression smears	Eggs
10	Pinheiro et al. (2018)	*Cerdocyon thous*	Brazil	*Capillaria* sp*.*	SEM, Microscopy and Histology	Adults and Eggs
11	Masuda et al. (2019)	*Sus scrofa*	Japan	*Capillaria* sp*.*	Histology and Molecular	Adults and Eggs
12	Rothenburguer et al. (2019)	*Rattus norvegicus*	Canada	*Eucoleus* sp*.*	Necropsy	Adults and Eggs
13	Pacifico et al. (2022)	*Sus scrofa*	Italy	*Eucoleus garfiai*	Molecular and Histology	Adults
14	Power et al. (2023)	*Sus scrofa*	Italy	*Eucoleus garfiai*	Molecular and Histology	Adults
15	Lombardo et al. (2023)	*Procyon lotor*	Italy	*Capillaria procyonis*	Histology	Adults and Eggs
16	Tamaru et al. (2025)	*Sus scrofa*	Japan	*Eucoleus garfiai*	Molecular, SEM, Microscopy	Adults
17	Breidahl et al. (2025)	*Isoodon obesulus*	Australia	*Eucoleus longiductus*	Necropsy and Histology	Adults
18	Gürel & Umur (2026)	*Sus scrofa*	Türkiye	*Eucoleus garfiai*	Microscopy Molecular and Histology	Adults

Over the years, histological techniques have been frequently used to diagnose parasite species. However, when these are isolated, there are cases where only genus-level characterization of the helminth's taxonomy is possible. With the advent of molecular tests, species-level identification has become more effective, although histology is still used as a complement and for the analysis of tissue lesions caused by the parasite-host relationship.

## Discussion

### Prevalence

The adaptability of Capillariidae is evidenced in aquatic environments, as seen in the work of Van Banning (1980), who reported the occurrence of black spots in *Cynoglossus browni* in Senegal, which was associated with eggs of *Capillaria spinosa*. This parasite was previously described in *Carcharhinus milberti*, which suggest a link between various fish in the life cycle of the capillariid.

In mammals, a high prevalence of Capillariidae in *Procyon lotor* is remarkably high in the United States. In Illinois, Snyder (1988) demonstrated a prevalence of 90% of *Capillaria procyonis* eggs in tissues, indicating that this is a primary site for infection and that capillariosis is endemic and extremely common in this population. Subsequently, Snyder (1989), provided more details on the exact location of the parasite in this region of the host. In the eastern US, Hamir & Rupprechtt (1998) point out this although there is an overall prevalence of 45%, that is still a high infection rate. They locate the parasite in the oral mucosa, esophagus, and urinary bladder. However, these infections are generally considered to have low pathogenicity and are characterized by tunnels in the epithelium.

The study by Hamir & Snyder (1999) also investigated capillariosis in *Procyon lotor* in Oregon, contributing to the knowledge of the parasite's distribution. Likewise, Tamaru et al. (2025) documented the detection of endoparasites, including Capillariidae, in non-native *P. lotor* in Central Italy, extending the relevance of the infection the European fauna.

In *Sus scrofa*, the most relevant species is *Eucoleus garfiai*, according to Pacifico et al. (2022). They recorded for the first time in Italy a high prevalence in these animals, with the parasite detected in the gastric and lingual mucosa. Furthermore, a subsequent epidemiological study by Power et al. (2023) observed that the prevalence of *E. garfiai* was significantly higher at high elevations, reaching 66.67%, a finding that suggests a direct link with the greater presence of intermediate hosts (earthworms) in better quality soils in these areas.

However, the distribution of *E. garfiai* is not limited to Europe. Masuda et al. (2019) confirmed its presence in the lingual mucosa of wild *S. scrofa* in Japan, marking the first record outside the European continent and highlighting the cosmopolitan distribution of the infection in swine. However, it was in 2026 that Gürel & Umur (2026) reported a prevalence of 83%, the highest to date for this species in swine in Turkey and worldwide, confirmed by molecular and morphological data.

The location in the tongue was also already noted in domestic pigs in Papua New Guinea, by a new species, *Capillaria papuensis*, also in the epithelium of the tongue, with minimal pathology, as seen in the study by Copland, (1975). Furthermore, the pattern of infection on the tongue is a recurring characteristic of capillariosis in carnivores and omnivores. Although the sample was limited, the detection of Capillariidae on the tongue of the *Cerdocyon thous* in Pará, Brazil, establishes an important neotropical data point (2018). In birds of prey, Royal & Jochems (2018) investigated a lesion on the tongue of a Great Horned Owl that strongly suggesting etiology by Capillariidae.

In birds of prey in Türkiye, Yildiz et al. (2015) report the first case of *Eucoleus dispar* in *Buteo buteo*, with the nematode and its eggs being observed in the epithelium of the tongue. Previously, in urban rodents in Canada, Rothenburger et al. (2014) found a high prevalence of 73.8% in *Rattus norvegicus* presenting infection or lesions associated with *Eucoleus* sp. in the non-glandular stomach, with a prevalence of infection of 43.1% in the upper gastrointestinal tract. The study by Rothenburger et al. (2019) deepened the knowledge about the pathology in these urban rats in Vancouver. Finally, a new host was added to the spectrum of studies, investigating the causes of morbidity and mortality in an endangered marsupial, *Isoodon obesulus*, possibly finding infections by Capillariidae (2025).

### Hosts

The prevalence of the Capillariidae family is remarkably high in free-living hosts worldwide, with its epidemiology strongly influenced by feeding habits and the parasite's complex life cycle, which, although unknown in many hosts, frequently involves intermediate hosts such as oligochaetes. In terrestrial mammals, the high prevalence is apparently directly linked to omnivory and foraging on the ground. In the United States, Snyder (1988) reported that *Procyon lotor* exhibits endemic rates, with *Capillaria procyonis* eggs detected on 90% of tongues, while other species occupy niches such as the urinary bladder, according to Hamir & Snyder (1999). Studies by Hamir & Rupprechtt (1998) and Hamir & Snyder (1999) on *P. lotor* in the USA provide concrete evidence of the parasite's predilection for different anatomical sites within this host.

Similarly, infection by *Eucoleus garfiai* in *Sus scrofa* in Italy and Japan reflects the ingestion of earthworms during foraging. The prevalence demonstrates how soil quality and the density of intermediate hosts drive the Italian cycle, as discussed by Power et al. (2023). This tropism for the upper alimentary tract is also seen in canids in Brazil and in birds of prey in Türkiye, as described in the studies by Pinheiro et al. (2018) and Yildiz et al. (2015), respectively, reinforcing the oral transmission mechanism.

The adaptability of Capillariidae extends to the aquatic environment. The detection of eggs of *Capillaria spinosa*, a parasite originally described in *Cynoglossus browni*, illustrates the adaptability and complex life cycles that involve the marine food (1980). Whether in terrestrial or aquatic hosts, the prevalence of capillariosis reflects the ecological success of the parasite in exploiting the feeding pathways of hosts from different classes, resulting in chronic and widely distributed infections.

### Types of technical analyses

Historically, the most widely used method for identifying capillariosis is the morphological analysis of the eggs. These eggs are characteristically barrel-shaped or oval, with bipolar plugs (2018). Identification in mammals is based on the detection of these eggs in different fluids or tissues. For example, the high prevalence of *Capillaria procyonis* in raccoons was established by Snyder (1988) through the routine finding of characteristic eggs in the sediment of acidic digests of the tongue, a method that allowed quantifying the infection in an unusual site. The search for new information on the location of the parasite (1989) was also based on exfoliation and visualization techniques.

Histopathology has become the technique of excellence for confirming infections in tissues with embedded parasites, such as the tongue and stomach. Pinheiro et al. (2018), when studying *Cerdocyon thous* in Brazil, based their diagnosis on the analysis of histological sections of the tongue, which showed adult nematodes embedded in the epithelium and, crucially, the presence of eggs inside the females, confirming the family Capillariidae and the genus *Capillaria* (also using scanning electron microscopy for superficial details). Similarly, the description of *Capillaria papuensis* in pigs by Copland (1975) and the studies of *Eucoleus dispar* in birds by Yildiz et al. (2015) also made extensive use of histology to confirm the intramucosal location of adult worms and eggs. In wild boars, histopathology was essential for confirming the location of *Eucoleus garfiai* in the gastric and lingual mucosa (2022). The finding in rats by Rothenburger et al. (2014, 2019) also relied heavily on histopathology to describe associated lesions.

More recently, molecular biology techniques (PCR and sequencing) have complemented morphological diagnosis. In the most recent studies on *Eucoleus garfiai* in *Sus scrofa* in Japan, Italy, and Turkey, Masuda et al. (2019), Power et al. (2023), and Gürel & Umur (2026) combined histopathological analysis and molecular analysis (PCR), which allowed them not only to identify the parasite (2019) and to study of prevalence, but confirmed the species identity by phylogeny, a level of precision that transcends the limitation of simple egg morphology. Integrated taxonomic approaches, as described by Lombardo et al. (2023), represent the state of the art in the accurate identification of these nematodes.

## Conclusion

The analysis of the prevalence, ecology, and diagnosis of the Capillariidae family reveals a group of nematodes with remarkable ecological success, characterized by their wide global distribution and extreme adaptability to a variety of hosts (mammals, birds, and fish) and niches. The high prevalence in omnivorous and foraging hosts is intrinsically linked to the ingestion of intermediate hosts, and demonstrates a strong connection between the parasite's ecology and the host's feeding habits. Genera such as *Capillaria* and *Eucoleus* demonstrate a marked tropism for the upper alimentary tract and, in less common cases, for the urinary tract, illustrating the diversity of niches within the family.

Diagnosis has evolved from the classic morphology of bipolar eggs and histopathology, which are still used for parasite-host characterization, to integration with molecular biology. This integrated approach is fundamental for enabling a definitive confirmation of the species and a precise understanding of the epidemiology and zoonotic potential of these widely distributed parasites. In summary, capillariosis, although often underestimated, represents an important marker of wildlife health and ecological interactions, meaning that further studies on the subject will be necessary.
